# A Computational Inter-Species Study on Safrole Phase I Metabolism-Dependent Bioactivation: A Mechanistic Insight into the Study of Possible Differences among Species

**DOI:** 10.3390/toxins15020094

**Published:** 2023-01-18

**Authors:** Lorenzo Pedroni, Jochem Louisse, Ans Punt, Jean Lou C. M. Dorne, Chiara Dall’Asta, Luca Dellafiora

**Affiliations:** 1 Department of Food and Drug, University of Parma, 43124 Parma, Italy; 2 Wageningen Food Safety Research, P.O. Box 230, 6700 AE Wageningen, The Netherlands; 3 Methodology and Scientific Support Unit (MESE), European Food Safety Authority, 43126 Parma, Italy

**Keywords:** alkenylbenzenes, cytochrome P450, safrole, estragole, CYP1A2, CYP2A6, molecular modeling

## Abstract

Safrole, a 162.2 Da natural compound belonging to the alkenylbenzenes class, is classified as a possible carcinogen to humans by IARC (group IIB) and has proven to be genotoxic and carcinogenic to rodents. Despite its use as a food or feed additive, it is forbidden in many countries due to its documented toxicity; yet, it is still broadly present within food and feed and is particularly abundant in spices, herbs and essential oils. Specifically, safrole may exert its toxicity upon bioactivation to its proximate carcinogen 1′-hydroxy-safrole via specific members of the cytochrome P450 protein family with a certain inter/intra-species variability. To investigate this variability, an in-silico workflow based on molecular modelling, docking and molecular dynamics has been successfully applied. This work highlighted the mechanistic basis underpinning differences among humans, cats, chickens, goats, sheep, dogs, mice, pigs, rats and rabbits. The chosen metric to estimate the likeliness of formation of 1′-hydroxy-safrole by the species-specific cytochrome P450 under investigation allowed for the provision of a knowledge-based ground to rationally design and prioritise further experiments and deepen the current understanding of alkenylbenzenes bioactivation and CYPs mechanics. Both are crucial for a more informed framework of analysis for safrole toxicity.

## 1. Introduction

**Figure 1:**
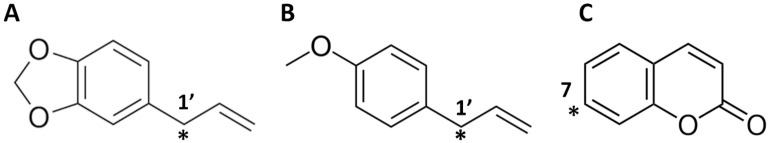
Chemical structure of molecules under analysis. The asterisk indicates the atom undergoing the reaction. (**A**) Safrole; (**B**) Estragole; (**C**) Coumarin.

Safrole (Figure 1) is a natural low-molecular-weight molecule (162.2 Da) belonging to alkenylbenzenes [1]. Alkenylbenzenes are secondary metabolites of herbs and spices (e.g., basil, fennel and parsley) that are commonly abundant in essential oils, with many members of interest for their potential adverse health effects, including safrole [2]. It has been reported that some alkenylbenzenes are carcinogenic via a genotoxic mode of action, through their highly reactive phase II 1′-sulfooxy metabolites [3]. Specifically, safrole may exert its toxicity upon its conversion to 1′-hydroxy-safrole (a proximate carcinogen) due to the cytochrome P450 (CYP)-dependent hydroxylation formed in humans mainly by CYP2A6 and subsequent sulfation to form 1′sulfooxy-safrole (the ultimate carcinogen). The 1′-sulfooxy metabolite is responsible for the formation of DNA adducts causing the genotoxic insult [4]. Moreover, animal studies have suggested that safrole may cause tumors in liver and other tissues in vivo, and IARC classified safrole as possibly carcinogenic to humans (group IIB) [1,5].

The use of safrole as a food or feed additive is no longer allowed in many countries due to its documented toxicity (e.g., its use has been banned in the US since the 1960s). However, it is still present in food and feed due to its abundance in spices, herbs and essential oils used as ingredients, giving rise to multiple safety issues [6,7,8]. Safrole has been found in a variety of food products including cola-tasting beverages and Bologna and Vienna sausages to cite but a few, and it can also be found in plant-based foods, food supplements and feed/food products with safrole-containing essential oils added as ingredients [2,6,9,10].

Safrole toxicity has been proven in animal models, although a certain inter-species variability has been documented. This discrepancy in the data raised the question of whether the genotoxic effects observed in experimental animals are also expectable in humans [11] or other animal species, including farm and companion animals. In line with this interpretation, a certain degree of variability is likely expected among various animal species. As the toxicity of safrole and other alkenylbenzenes depends on its bioactivation of 1′-hydroxy-safrole, inter- and intra-species differences in biotransformation are of interest. In fact, differences in terms of 1′-hydroxy-safrole formation are expected to diversify inter- and intra-species sensitivity to safrole. Indeed, many in vitro studies have been performed with human and rat liver fractions to study the bioactivation and detoxification kinetics of various alkenylbenzenes [12]. Besides these in vitro studies, it would be of interest to obtain further insight into inter- and intra-species differences in bioactivation via molecular modelling studies. Furthermore, there are limited data available on the biotransformation of alkenlylbenzenes in animals other than mice and rats, and the existing in vitro data are only for rats and humans. This indicates a knowledge gap regarding the bioactivation potential of alkenylbenzenes in other species, including farm and companion animals, which may be at risk from consuming feed with safrole-containing ingredients. Nonetheless, such knowledge is important both to ensure better feed safety and animal welfare and to drive a more informed selection of animal models for data extrapolation to humans. However, the systematic study of livestock animals and pets is hampered by the high costs of such studies and related ethical issues. In this sense, the use of new approach methodologies (NAMs) may provide alternative and effective methods of analysis. NAMs include in vitro methods to study the biotransformation of chemicals, but these generally require primary animal material and can be expensive for large-scale analysis due to, for example, the extensive chemical analytical measurements required. NAMs also include a variety of non-testing approaches, including computational methodologies, with increasing use in the evaluation of chemical safety and, more generally, within the framework of next-generation risk assessments [13]. Regarding in silico biotransformation studies, 3D molecular modelling techniques have already been applied to study the biotransformation of small molecules by CYPs, providing a useful means of distinguishing substrates from non-substrates [14,15,16]. Specifically, molecular dynamics at a low nanosecond scale (<50 nanoseconds) applying the same method used in this study has already succeeded in predicting the likeliness of small molecules to be substrates of CYPs [15]. Therefore, this previously validated 3D modelling framework based on docking studies and molecular dynamics simulations is applied in the present study to assess the CYP-dependent capability of nine animal species including farm and companion animals (i.e., rat, mouse, dog, cat, rabbit, pig, goat, sheep and chicken) to form the safrole proximate carcinogen 1′-hydroxy-safrole. Such information could provide insights into possible species differences in bioactivation and related susceptibility to safrole-dependent genotoxicity, supplying a blueprint to (i) informedly design further experimental trials; (ii) refine other knowledge-based modelling techniques; and (iii) expand the current understanding of safrole toxicity and bioactivation and CYPs mechanics. More specifically, the present study assessed the dynamics of the interactions of the alkenylbenzenes safrole and estragole with human CYP1A2 and CYP2A6. These CYPs are reported to be involved in the 1′-hydroxylation of various alkenylbenzenes [4] (safrole by CYP2A6, estragole by CYP1A2 and CYP2A6) and coumarin, which is reported to be a substrate that is detoxified (to 7-hydroxycoumarin) by CYP2A6 [17]. Subsequently, molecular dynamics were used to study the interactions between the alkenylbenzenes and homologs of CYP1A2 and CYP2A6 in other species (rat, mouse, dog, cat, rabbit, pig, goat, sheep and chicken), estimating the capability of these species to bioactivate safrole to its proximate carcinogen 1′-hydroxy-safrole.

## 2. Results and Discussion

In a previous study, we demonstrated that computing the distance between the atom undergoing the reaction of small molecules and the Fe atom of the heme group over time can be used as a reliable geometrical criterion to distinguish CYP substrates from non-substrate molecules [15]. The same workflow has been applied here to study the substrate likeliness of safrole for CYP1A2 and CYP2A6 in a set of animal species including rats, mice, dogs, cats, rabbits, pigs, goats, sheep and chickens. For this purpose, the primary sequence of each CYP under analysis was downloaded in the FASTA format to generate 3D models via homology modelling (see Materials and Methods and Supplementary Materials for further details). Before the analysis, the actual expression of the considered CYPs in the liver of animals under analysis has been checked by browsing reference databases of protein existence (i.e., UniProt; https://www.uniprot.org accessed on 10 January 2023) and gene transcription and expression (i.e., Expression Atlas, https://www.ebi.ac.uk/gxa/home [18] accessed on 10 January 2023; Bgee, https://bgee.org [19] accessed on 10 January 2023). For all of them, at least one piece of evidence for existence and expression in the liver, which is the metabolizing organ relevant to safrole toxicity, has been found (Table S1; Supporting Material).

Once the actual hepatic expression of CYPs under analysis had been ascertained, the binding architecture of ligands was calculated via docking simulations and the geometry of binding was analysed over time with molecular dynamics. The binding poses produced by docking analysis describing the interactions of safrole with amino acids forming the binding site and its orientation to the Fe heme are reported in Supplementary Materials (Figures S1–S3). Specifically, safrole showed similar binding poses in all the calculated complexes with CYP2A6 or CYP1A2 homologs, where the atom undergoing the reaction was directed to the heme Fe. The contribution of hydrophobic/hydrophobic interactions was found to be predominant in all the safrole-CYP complexes, as shown in Figures S1–S3 (Supporting Materials). The formation of hydrogen bonds was observed in certain complexes (i.e., human, cat, pig, rabbit, chicken and rat homologs of CYP1A2 and in human, cat, pig and sheep homologs of CYP2A6). However, the hydrophobic/hydrophobic interactions were expected to be crucial based on the marked hydrophobicity of safrole (LogP 3.45, as per PubChem database; PubChem CID 5144, last accessed 24 November 2022). Although safrole was found to be comparable among the various complexes generated by docking, the arrangement of the atom undergoing the reaction with the heme Fe was found to vary over time in certain complexes. Specifically, the complexes where the distance to the heme Fe increased over a certain threshold were predicted not likely to lead to 1′-hydroxy-safrole formation (see below).

### 2.1. Fit-for-Purpose Validation

Once the CYP models had been derived, a fit-for-purpose validation was carried out to assure the applicability to CYP1A2 and CYP2A6 of the procedure and related threshold that previously succeeded with CYP2D6 [15]. The validation relied on data retrieved from the literature describing the capability of safrole to be biotransformed to 1′hydroxy-safrole by human CYP2A6, while its conversion by human CYP1A2 was found to be negligible [4]. The safrole congener estragole (Figure 1) was also included, being a substrate of human CYP1A2 and CYP2A6 [4], along with coumarin (Figure 1) as an additional positive control. In fact, coumarin is a substrate of human CYP2A6 and rat CYP2A3 (the proximate homolog of human CYP2A6) [17]. Therefore, the interaction of safrole and estragole was computed in human CYP1A2 and CYP2A6, while the interaction of coumarin was computed in human CYP2A6 and rat CYP2A3 to challenge the geometrical criteria and threshold previously described to infer the substrate likeliness of ligands [15]. In more detail, in agreement with a previous study [15], the distance between the atom undergoing the reaction and the heme’s Fe can be monitored to qualitatively distinguish CYP substrates from non-substrate molecules. Based on previous evidence [15], an average interatomic distance of 0.53 nm was set as the threshold and molecules whose atoms undergoing the reaction show a wider distance could be considered to not be efficiently biotransformed at that specific position.

**Figure 2:**
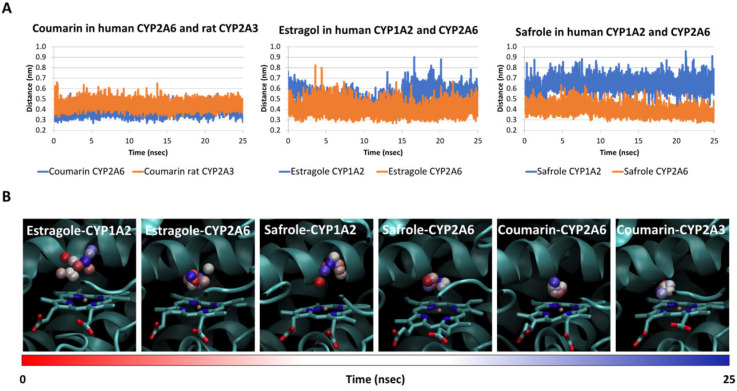
Molecular dynamics results of estragole and safrole in complexes with human CYP1A2 and CYP2D6 and coumarin in complexes with human CYP2A6 or rat CYP2A3. (**A**) Analysis of the distance between heme’s Fe and the atom undergoing the reaction and of coumarin in human CYP2A6 and rat CYP2A3, estragole in human CYP1A2 and CYP2A6, and safrole in human CYP1A2 and CY2A6. (**B**) Trajectory analysis of the complexes mentioned above. The proteins are represented by cartoons, while heme is represented by sticks. The atom undergoing the reaction is represented by spheres and a red-to-blue colour change indicates the stepwise changes of coordinates over time.

Therefore, the distance between the heme’s Fe and the atom undergoing the reaction of estragole in CYP1A2 and CYP2A6, that of safrole in CYP2A6, and that of coumarin in CYP2A6 and rat CYP2A3 were expected to be lower compared to that of the safrole-CYP1A2 complex, for which a lack of biotransformation has been demonstrated [4]. The initial architectures of binding were computed via docking simulations. As shown in Table 1, docking scores were all positive and relatively high, suggesting the theoretical capability of the molecules under analysis to appreciably interact with the CYP’s catalytic site, in agreement with previous studies (the higher the score, the better the fitting into the model’s pocket). Therefore, substrates could not be distinguished from non-substrate molecules based on docking scores alone. However, we previously showed that dynamic simulations should be combined with docking studies to better estimate the likeliness of CYPs substrates [15]. As shown in Figure 2, both the interatomic distance analysis and the inspection of trajectories over time supported this assumption as the atom undergoing the reaction was kept close to the heme’s Fe only in complexes with an appreciable biotransformation capacity that had been previously demonstrated in vitro.

Jeurissen and co-workers reported that estragole is a better substrate for human CYP2A6 than for CYP1A2, with Kcat/Km values of 341 and 59, respectively [4]. As shown in Figure 2A, the interatomic distance between the atom undergoing the reaction of estragole and the heme’s Fe was similar for CYP1A2 and CYP2A6 up to 15 nsec while moving away, with a significantly larger mean distance for CYP1A2 compared to CYP2A6 (0.51 ± 0.09 and 0.40 ± 0.06 nm, respectively; *p* < 0.001). Considering that the proximity between the atom undergoing the reaction and heme’s Fe is fundamental to ensure the reaction (see above), the transformation by CYP1A2 was expected to be less effective compared to CYP2A6, in agreement with in vitro experimental results. Additionally, the analysis of trajectories (Figure 2B) revealed a spread of the atom undergoing the reaction that was wider when estragole was in a complex with CYP1A2 than when it was in a complex with CYP2A6. In line with this interpretation, safrole was described as a substrate of CYP2A6 (with Kcat/Km of 160) but not of CYP1A2 (no biotransformation), as reported by Jeurissen et al. [4]. Both the computed distances and trajectories are in line with this evidence as the interatomic distances were significantly different in complexes with CYP1A2 or CYP2A6 (0.63 ± 0.06 and 0.40 ± 0.04 nm, respectively; *p* < 0.001). Furthermore, the analysis of trajectories showed a wider spread of the atom undergoing the reaction when in a complex with CYP1A2 than when in a complex with CYP2A6 (Figure 2B). As an additional control, coumarin was also included in the analysis and its interaction was calculated with human CYP2A6 and rat CYP2A3, which showed a comparable in vitro reaction yield, as previously reported [17]. As shown in Figure 2, the interatomic distance (0.38 ± 0.04 and 0.44 ± 0.04 nm with CYP2A6 and rat CYP2A3, respectively) and the spread of the atom undergoing the reaction to the heme’s Fe in the two coumarin-CYP complexes were similar. Taken together, these results further supported the reliability of the modelling framework to predict whether chemicals are substrates or not for human CYP1A2 and CYP2A6 and animal homologues. The analysis of distances over time confirmed the reliability of the mean distance threshold that was previously identified and set at 0.53 nm. Of note, these data might suggest that a (semi)quantitative analysis is possible considering that the computed distances of estragole were consistent with the rank of the two CYPs in terms of biotransformation yield (the better the CYP to transform estragole, the shorter the computed interatomic distance and the lower the spread of the atom undergoing the reaction within the catalytic site). However, a wide set of positive and negative controls structurally related to the molecules under investigation are needed to properly validate the model for quantitative purposes, which could not be found at the time of analysis. Therefore, the model was meant to provide qualitative predictions.

### 2.2. Interaction of Safrole with Animal Homologs of Human CYP1A2 and CYP2A6

The 3D models of animal homologs of human CYP1A2 and CYP2A6 were derived via homology modelling in agreement with a previous study [20] and carried forth to docking analysis and molecular dynamic simulations to investigate the interaction with safrole over time and estimate its substrate likeliness to form 1′-hydroxy-safrole [15].

As per CYP2A6, only sheep had a cytochrome entry referred to as CYP2A6. For the other species, the proximate homolog to human CYP2A6, i.e., the animal’s CYP with the highest identity percentage to human CYP2A6, was searched for in the respective proteome to identify the CYP theoretically responsible for the safrole biotransformation in the animal species under investigation. Otherwise stated, it was assumed that the CYP that was most likely responsible for the potential biotransformation of safrole in each animal was the one that was most similar in terms of % identity to human CYP2A6. The search was conducted using the blastp tool of Web BLAST (Basic Local Alignment Search Tool) (https://blast.ncbi.nlm.nih.gov/Blast.cgi, accessed on 20 September 2022; see Materials and Methods for further details) [21]. As shown in Table S2 (Supplementary Materials), according to the blastp algorithm, at least one CYP with a very high percentage of sequence identity (above 80%) to human CYP2A6 was found in each species. As an exception, in the chicken proteome, the CYP most similar to the human homolog showed less than 50% of identity and was therefore deemed too divergent to preserve significant functional conservation in terms of its capability to form 1′-hydroxy-safrole. Therefore, it was not further analysed. Once the accession numbers of all the proximate homologs had been identified, their primary sequence was collected in FASTA format and used to derive the respective 3D model via homology modelling (see Materials and Methods for further details). For rabbits, two homologs (CYP2A10 and CYP2A11) were carried forth to the analysis as they showed the same identity percentage to human CYP2A6.

**Figure 3:**
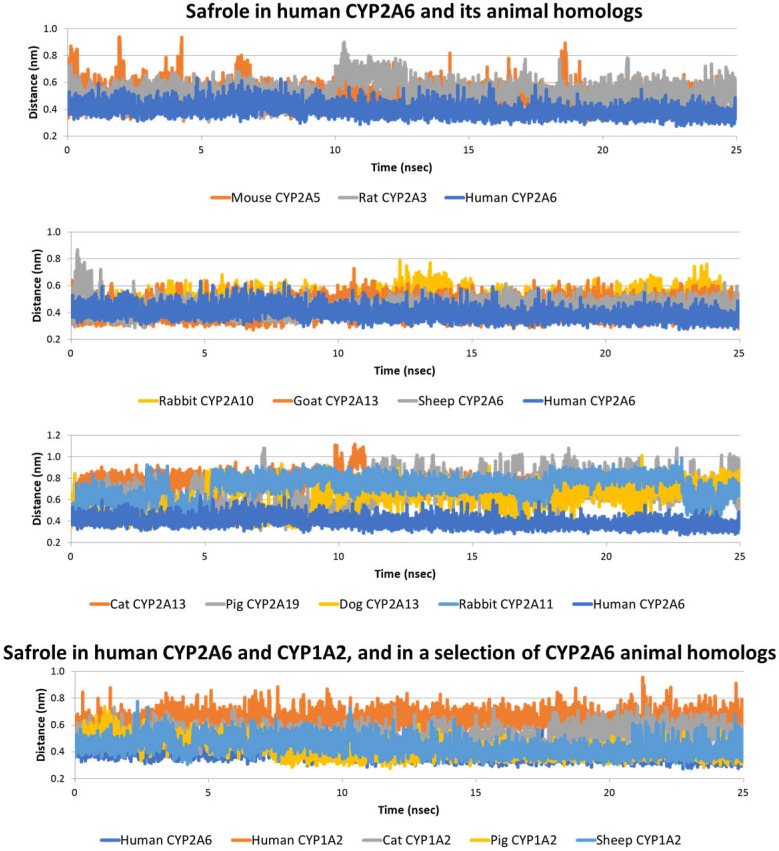
Molecular dynamics results of safrole in complexes with human CYP1A2 and CYP2A6 and a selection of CYP1A2 and CYP2A6 animal homologs.

Each safrole-CYP complex showed a high docking score, suggesting the theoretical capability of safrole to favourably interact with all the animal homologs under analysis. Molecular dynamics simulations were then analysed to verify this assumption. As shown in Figure 3 and Table 2, the interaction of safrole with the rat and mouse homologs showed a dynamic of interaction similar to that found for the human CYP2A6, although in both species, the distance between the atom undergoing the reaction and the heme’s Fe was larger compared to the human case. This could suggest that safrole might be less efficiently biotransformed to the 1’-hydroxymetabolite by rat and mouse homologs compared to human CYP2A6.

This result is in line with previous in vitro evidence describing the lower activity of rat hepatic microsomes compared to human liver microsomes to form 1′-hydroxy-safrole [12,23] and may propose a plausible mechanistic explanation (which could be also expected for mice). However, a diverse level of expression of human CYP2A6 and rat CYP2A3 in liver tissues could have a role in diversifying the formation of 1′-hydroxy-safrole in the two species as the hepatic expression of CYP2A3 in rats has been documented but not quantitatively compared to that of humans [24]. Nonetheless, the agreement between the computational results and the evidence previously collected (see above), along with the data collected for coumarin (see above; Section 2.1), further support the reliability of the procedure and suggest that these in silico analyses can provide important insights for other animal homologs. As shown in Figure 3 and Table 2, safrole in sheep CYP2A6, goat CYP2A13 and rabbit 2A10 showed dynamics of interactions, with interatomic distances between its atoms undergoing the reaction and the heme’s Fe, which were similar to that observed for human CYP2A6. This suggested that sheep CYP2A6, goat CYP2A13 and rabbit CYP2A10 may appreciably form 1′-hydroxy-safrole. It must be noted that, although the in silico analysis suggested safrole bioactivation, data for protein expression, which is, of course, required for bioactivation to occur, are still fragmentary and require further dedicated investigations. Conversely, the CYP2A6 homologs identified in cats, dogs and pigs and the 2nd homolog identified in rabbits (CYP2A11) showed a dynamic of interaction not in line with an appreciable formation of 1′-hydroxy-safrole. Indeed, the interatomic distances between the atom undergoing the reaction and the heme’s Fe were similar to that observed for the human safrole-CYP1A2 complex (i.e., not showing biotransformation in vitro [4]) and above the 0.53 nm threshold. Although the existence of other CYP2A6 homologs able to form 1′hydroxy-safrole cannot be excluded completely, our results suggest that for cats, dogs and pigs, a much less efficient formation of the proximate carcinogen can be expected, which may possibly translate to lower degrees of safrole toxicity. This cannot be concluded from the present in silico analysis as a CYP2A6 homolog-independent biotransformation may exist, but this study provides clear directions for further dedicated (in vitro) investigations.

Concerning the animal homologs of human CYP1A2, all the species under investigation (i.e., rat, mouse, dog, cat, rabbit, pig, goat, sheep and chicken) had in the respective proteome recorded in the NCBI Entrez database (https://www.ncbi.nlm.nih.gov/search accessed on 20 September 2022) [25] a cytochrome annotated as CYP1A2 (Table S2; Supplementary Materials). Each sequence was downloaded in the FASTA format and used to derive a 3D model via homology modelling (see Materials and Methods for further details). As shown in Table 1, all the docking scores were again relatively high, suggesting the theoretical capability of safrole to interact with all of them. Each complex was then analysed via molecular dynamics to monitor the geometrical stability and respective orientation of the atom undergoing the reaction to the heme’s Fe. Surprisingly, as shown in Table 2 and Figure 3, all the animal homologs showed a better arrangement of safrole compared to the complexes with human CYP1A2 (i.e., the atom undergoing the reaction was closer to the heme’s Fe compared to safrole within human CYP1A2 and below the threshold of 0.53 nm). This may suggest that a certain degree of safrole biotransformation to 1′hydroxy-safrole could be expected for all the species analysed, even if the respective CYP2A6 homolog is inactive or with low biotransformation activity. It is of interest that the cat homolog showed the widest mean interatomic distance and an interatomic distance trend resembling that observed for human CYP1A2. This could suggest a low capability of the cat CYP1A2 homolog to form 1′-hydroxy-safrole compared to the other species considered. This result, along with the evidence observed for the cat homolog of CYP2A6 (see above), might point to its possible low production of 1′hydroxy-safrole, which should be assessed in further experiments, along with the role of phase II reactions to better assess the associated sensitivity to the genotoxic effects of safrole.

### 2.3. Estimate of Binding Energy

The Prodigy Web Server [26] (https://wenmr.science.uu.nl/prodigy accessed on 18 November 2022) was used to estimate the binding free energy of each complex to study the safrole-CYP interaction beyond the capability to form the proximate carcinogen of safrole (i.e., 1′-hydroxy-safrole). To do so, a cluster analysis of each complex trajectory was performed first to obtain geometries of binding that are representative of the whole dynamics and using such poses as input for Prodigy (see Materials and Methods for further details). Of note, this approach was chosen for its actual applicability to an “in-bulk” analysis (24 complexes analysed), which is often not affordable in terms of computational efforts using other MD-based approaches. Moreover, this would have been beyond the scope of our work considering that the binding-free energy is not related directly to the likelihood of a substrate being converted into a specific product.

From the analysis, all the protein-ligand complexes were found to be energetically favoured (the calculated free energy ranged from –7.4 to –7.0 Kcal/mol; Supplementary Materials, Table S6) suggesting that all the CYPs analysed may favourably interact with safrole (in line with docking analysis; see above). However, the geometry of binding was not always compatible with the formation of the 1′-hydroxy-safrole, although the formation of other metabolites could not be excluded. Therefore, the interaction between safrole and the CYPs under analysis was hypothesised as likely to occur with all the CYPs analysed, but the formation of safrole proximate carcinogen (i.e., 1′-hydroxy-safrole) may occur on a species-specific basis.

## 3. Conclusions

This study demonstrated the effectiveness of the proposed modelling framework to predict the likelihood of small molecules being metabolised by members of the CYP enzyme family. The distance computed over time between the atom undergoing the reaction and the heme’s Fe could be used as a geometric rationale to estimate the likeliness of the formation of a given metabolite.

Concerning the case studies presented, the evidence collected provides a knowledge-based ground that is useful to rationally design further experiments, such as a focused analysis of the hepatic expression of CYP enzymes in different species and the kinetics of biotransformation for specific CYP isoforms, to cite but a few. This is an essential piece of information to characterise species-specific differences in terms of biotransformation, bioactivation and related toxicity. In this respect, specific CYP isoforms have been mined from animal proteomes and prioritised for future assessment. Specifically, all the species considered (rat, mouse, dog, cat, rabbit, pig, goat, sheep and chicken) seemed prone to bioactivate safrole, having at least one homolog of CYP1A2 or CYP2A6 theoretically able to form 1′hydroxy-safrole. In addition, all the complexes analysed were found to be energetically stable, suggesting the possible capability of the CYPs under analysis to interact with safrole and biotransform it. However, the production of safrole’s proximate carcinogen (i.e., 1′-hydroy-safrole) has been found to differ between species. Cats seem to be the species less likely to bioactivate safrole, considering the calculated lack of formation of 1′hydroxy-safrole by its CYP2A6 homolog (CYP2A13) and the low biotransformation yield expected for its CYP1A2. Nonetheless, the existence in cat proteome of other CYPs able to significantly form 1′hydroxy-safrole cannot be excluded. Additionally, the relatively high hepatic expression of cat CYPs reported in the Bgee database (Table S1; Supporting Material), which is in line with the expression reported for human homologs, may eventually lead to significant biotransformation of safrole. Therefore, in safrole biotransformation studies using cat liver tissue fractions, the measurement of CYPs expression and activity, and the study of phase II reactions would be informative and aid in a better characterisation of cats’ susceptibility to safrole toxicity. This would be important from a feed safety perspective as cats have the lowest calculated safe concentrations for the presence of safrole-containing essential oils (22 mg/kg of complete feed) in feed reported thus far for farm and companion animals [10]. Conversely, sheep and goats, for which the highest calculated safe concentrations of safrole-containing essential oils in feed have been proposed (50 mg/kg; EFSA, 2022), appeared to have the highest performing CYP homologs in terms of forming 1′hydroxy-safrole. However, according to the Bgee database, sheep have the lowest hepatic expression scores for the considered CYPs, possibly suggesting reduced biotransformation due to their limited expression at the main metabolizing organ for safrole toxicity (see Table S1; Supporting Material). Therefore, information on the actual hepatic expression of CYP1A2 in both species and CYP2A13 and CYP2A6 in goat and sheep, respectively, along with a precise measurement of enzymatic activities, would be required to obtain better insights into the bioactivation capacities of these species. As the toxic effect depends on the formation of the reactive 1’sulfooxysafrole metabolite, (quantitative) information on other phase I and phase II reactions (integrated into physiologically-based kinetic (PBK) models) would be required to predict kinetic-dependent species differences in sensitivity to safrole toxicity. This will eventually provide a science-based approach to determine the safe use of safrole-containing essential oils for different species. From a general point of view, the results presented expand the current understanding of safrole toxicity and bioactivation and CYPs mechanics, which are both pivotal for a more informed framework of analysis to assess safrole toxicity. In addition, the outcomes described, when integrated either with experimental confirmations or other modelling techniques, may provide a useful line of evidence to support a more informed species extrapolation, helping the identification of animal species close to humans and valuable for the development of effective model systems.

## 4. Materials and Methods

### 4.1. Data Source

The 3D structures of safrole, estragole and coumarin were retrieved in .sdf format from the PubChem database (https://pubchem.ncbi.nlm.nih.gov accessed on 12 September 2022) [27] with CID 8815, 5144 and 323, respectively (CAS codes 140-67-0, 94-59-7 and 91-64-5, respectively). The models of human CYP1A2 and CYP2A6 were derived from the crystallographic structures recorded in the Protein Databank (PDB; https://www.rcsb.org accessed on 20 September 2022) [28] with PDB codes 2HI4 [29] and 2PG6 [30], respectively. In particular, the latter structure had two mutations, L240C and N297Q, which had reverted to the wild-type sequence, duly replacing the respective amino acid side chain using the Structure Editing/Rotamer tool of UCSF Chimera software (version 1.15) [31], as previously described [32]. The search for sequences of animal homologs to human CYP2A6 was conducted using the blastp tool of Web BLAST (Basic Local Alignment Search Tool) (https://blast.ncbi.nlm.nih.gov/Blast.cgi accessed on 20 September 2022) [21]. Default parameters were used with the sequence of human CYP2A6 as the input and searching in the non-redundant protein sequence database for each species under analysis. The sequence of animal homologs to human CYP1A2 and CYP2A6 were retrieved from the NCBI Entrez database (https://www.ncbi.nlm.nih.gov/search accessed on 20 September 2022) [25] and stored in FASTA format for further analysis (see below for further details). The accession numbers are reported in the Supplementary Materials (Table S3). The actual expression in the liver of the considered CYPs was checked by browsing references databases of protein existence (i.e., UniProt; https://www.uniprot.org/ accessed on 10 January 2023) and gene transcription and expression (i.e., Expression Atlas (https://www.ebi.ac.uk/gxa/home accessed on 10 January 2023) [18] and Bgee (https://bgee.org accessed on 10 January 2023) [19]). Specifically, UniProt provides information on protein in terms of protein/transcript existence and whether the protein has been inferred from homology. Expression Atlas and Bgee provide quantitative expression data at a protein or transcript level, with the latter specifically meant to provide data for intra-experiments quantitative comparisons [19].

### 4.2. Homology Modelling

**Figure 4:**
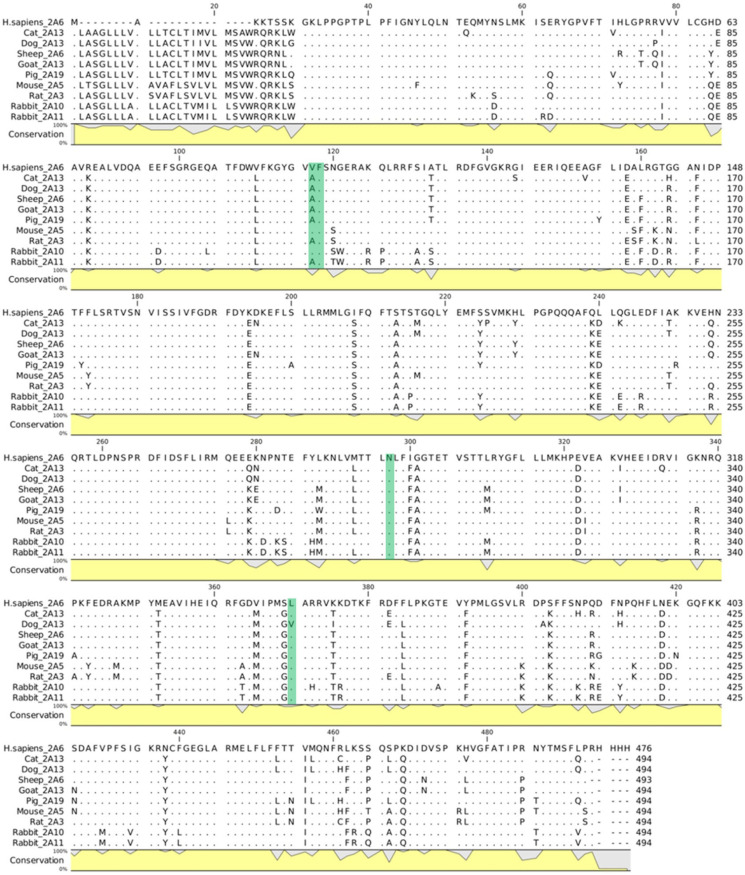
Multiple sequence alignment of animal homologs to human CYP2A6. Dots represent matching residues while dashes indicate gaps. The conservation line plot is reported in yellow and shows the percentage of conservation of each residue among the species with respect to the human sequence. The residues interacting with safrole in the human complexes are highlighted in green.

The structures of animal homologs of rat, mouse, dog, cat, rabbit, pig, goat, sheep and chicken to human CYP1A2 or CYP2A6 were not available in PDB at the time of analysis (last database accessed on 20 September 2022). Therefore, in agreement with previous studies [20,33], the respective models were derived via homology modelling using the human CYP1A2 or CYP2A6 structure as a template with the software Modeller (version 10.2) [34] interfaced in the UCSF Chimera software (version 1.16) [31]. Specifically, default settings were used apart from including non-water HETATM residues from templates to ensure the inclusion of the heme group in each model. The best-scored homolog model for each human CYP according to the ZDope scoring was carried forth to the analysis. Of note, we performed homology modelling via Modeller for continuity of the analysis with our previous study [15]. However, we compared the results obtained with those provided by AlphaFold2 [35], which has provided a significant breakthrough in structural biology. As shown in the Supplementary Materials, the legacy software Modeller and AlphaFold2 performed comparably, confirming the reliability of Modeller outputs. Indeed, the two sets of structures superimposed similarly to the human homolog with low RMSDs (see Supplementary Materials, Tables S4 and S5, and Figure S4). The percentage identity matrix and multiple sequence alignment proved the high identity among sequences and at the level of human residues interacting with safrole, and suggested the usability of human CYP as a reliable template for a canonical homology model procedure (see Figure 4 and Figure S5 and Supplementary Materials). The percentage identity matrices (PIMs; Supporting Materials, Figure S5) were conducted using Clustal Omega (https://www.ebi.ac.uk/Tools/msa/clustalo/ accessed on 18 November 2022) with default parameters [36], while the multiple sequence alignment was obtained using CLC Sequence Viewer version 7.7 (QIAGEN, Aarhus, Denmark; https://www.qiagenbioinformatics.com, accessed on 23 November 2022)

### 4.3. Docking Simulations

The docking analysis aimed to provide a plausible binding architecture for the molecules under analysis within the catalytic site of CYPs. This was performed with the GOLD software (version 2021), which has already shown high reliability for the evaluation of protein–ligand interactions, including in studies of the interactions with CYPs [15,33,37]. The binding site was defined within a 10-Å radius sphere around the centroid of the substrate binding site. The docking protocol was set according to previous studies, for which ligands were kept fully flexible and proteins semi-flexible, allowing polar hydrogens to rotate freely [15]. As a minor modification, the internal scoring function GOLDScore was used as it has been optimised for the prediction of binding architectures, according to the manufacturer declaration (https://www.ccdc.cam.ac.uk; accessed on 15 July 2022). In addition, the binding architecture of styrene—which has a structure closely related to that of estragole and safrole—and coumarin derived from a cytochrome’s crystallographic structures (PDB ID 4HGF and 1Z10, respectively) were used as a position restraint (constraint weight of 100 units) to facilitate the arrangement of safrole and estragole or coumarin, respectively. The pictures of binding poses (see Supporting Materials) were obtained through PLIP2021 (https://plip-tool.biotec.tu-dresden.de/ accessed on 18 November 2022) [38] and PyMol (version 2.3.0 Open-Source; https://pymol.org, accessed on 23 November 2022).

### 4.4. Molecular Dynamics

Molecular dynamics aimed to investigate the overall geometrical stability of CYP-ligand complexes over time to discriminate CYP substrates from non-substrate molecules. This was performed using GROMACS (version 2019.4) [39], while ligands were parametrised with the CHARMM27 all-atom force field [40]. The hydrogen database was modified according to previous works [41,42] to parametrise the heme group. Input structures were solvated with SPCE waters in a cubic periodic boundary condition, and counter ions (Na^+^ and Cl^−^) were added to neutralise the system. Prior to running simulations, each system was energetically minimised to avoid steric clashes and correct improper geometries using the steepest descent algorithm with a maximum of 5000 steps. Subsequently, each system underwent isothermal (300 K, coupling time 2 psec) and isobaric (1 bar, coupling time 2 psec) 100 psec simulations before running 25 nsec simulations (300 K with a coupling time of 0.1 psec and 1 bar with a coupling time of 2.0 psec).

### 4.5. Statistical Analysis

The statistical analysis of interatomic distances between the atom undergoing the reaction and the Fe atom of the heme group was conducted using SPSS IBM (v. 27.0, SPSS Inc., Chicago, IL, USA). For each complex, distance values of 5000 frames were considered, expressed as means ± standard deviation (SD) and compared to each other using one-way ANOVA (α = 0.05) with Bonferroni as a posthoc test (α = 0.05).

### 4.6. Cluster and Binding Affinity Analysis of Protein-Ligand Complex Trajectories

Each complex underwent a cluster analysis to identify geometries of binding representative of the whole simulation which were used to estimate the binding free energy. This was conducted using GROMACS (version 2019.4) [39] through the cluster command, using the gromos method and setting the cutoff at 0.3 nm, in agreement with previous studies [43]. The binding affinity was obtained by analysing the retrieved geometry of binding for each complex via the PRODIGY web application (https://wenmr.science.uu.nl/prodigy/lig accessed on 18 November 2022) [26].

## Figures and Tables

**Table 1:** Docking scores of estragole, coumarin and safrole within human CYP1A2 and CYP2A6 and their animal homologs.

Molecule	Species	CYP2A6 and its Homologues	CYP1A2
Estragole	Human	162	154
Coumarin	Rat	144	n.p.
	Human	146	n.p.
Safrole	Human	162	159
	Cat	174	153
	Chicken	n.p.	150
	Dog	164	152
	Goat	173	156
	Mouse	157	171
	Pig	172	155
	Rat	167	155
	Sheep	153	159
	Rabbit	174	161
		169	

Note: “n.p.” stands for “not performed”.

**Table 2:** Interatomic distance between the atom undergoing the reaction of estragole, coumarin or safrole and the heme’s Fe atom.

Molecule	Species	CYP2A6 and its Homologues		CYP1A2	
		Distance (nm)	Biotransformation ^1^	Distance (nm)	Biotransformation ^1^
Estragole	Human	0.40 ± 0.06	Yes (experimental) ^2^	0.51 ± 0.09	Yes (experimental) ^2^
Coumarin	Rat	0.45 ± 0.04	Yes (experimental) ^3^	n.p.	-
	Human	0.39 ± 0.04	Yes (experimental) ^3^	n.p.	-
Safrole	Human	0.39 ± 0.04	Yes (experimental) ^2^	0.64 ± 0.09	No (experimental) ^2, 4^
	Cat	0.74 ± 0.08	No (calculated)	0.52 ± 0.07	Yes (calculated)
	Dog	0.63 ± 0.14	No (calculated)	0.47 ± 0.06	Yes (calculated)
	Pig	0.65 ± 0.15	No (calculated)	0.43 ± 0.07	Yes (calculated)
	Goat	0.43 ± 0.07	Yes (calculated)	0.47 ± 0.06	Yes (calculated)
	Rabbit	0.72 ± 0.09	No (calculated)	0.47 ± 0.06	Yes (calculated)
		0.47 ± 0.08	Yes (calculated)		
	Chicken	n.p.	n.p.	0.47 ± 0.09	Yes (calculated)
	Sheep	0.43 ± 0.06	Yes (calculated)	0.44 ± 0.06	Yes (calculated)
	Mouse	0.50 ± 0.09	Yes (calculated)	0.48 ± 0.06	Yes (calculated)
	Rat	0.52 ± 0.08	Yes (calculated)	0.47 ± 0.06	Yes (calculated)

Note: Distances are expressed as mean values ± standard deviation. “n.p.” stands for “not performed”. ^1^ Refers to the capability to form 1′-hydroxy-metabolite of safrole and estragole or 7-hydroxy-coumarin from coumarin; ^2^ experimental evidence according to [4]; ^3^ experimental evidence according to [17]; ^4^ experimental evidence according to [22].

## Data Availability

Data are available upon request.

## Supplementary Materials

The following supporting information can be downloaded at https://www.mdpi.com/article/10.3390/toxins15020094/s1: Table S1: Expression data for considered CYPs at the liver level in the species under analysis; Table S2: Accession code and percentage identity of animal homologs to human CYP2A6; Table S3: Accession number for animal homolog to human CYP1A2 or CYP2A6; Table S4: Structural comparison between the CYP2A6 homology modelling structures obtained using Modeller and the AlphaFold Protein Structure Database; Table S5: Structural comparison between the CYP1A2 homology modelling structures obtained using Modeller and the AlphaFold Protein Structure Database; Table S6: Average predicted binding energy of the complexes under analysis; Figure S1: Binding architecture of safrole in complex with human CYP1A2 and its animal homologs. Protein is shown in white sticks, ligand in yellow and heme in green; Figure S2: Binding architecture of safrole in complex with human CYP2A6 and its animal homologs. Protein is shown in white sticks, ligand in yellow and heme in green; Figure S3: Binding architecture of estragole within human CYP1A2 and CYP2A6 (A) and coumarin in human CYP2A6 and rat CYP2A3 (B). Protein is shown in white sticks, ligand in yellow and heme in green; Figure S4: Structural alignment of the animals’ CYPs homologues over the human CYP2A6 (PDB ID 2PG6) and CYP1A2 (PDB ID 2HI4). A) CYP2A6 animal homologues structures obtained via the homology modelling procedure based on Modeller superimposed to the human CYP2A6 (PDB ID 2PG6). B) CYP2A6 animal homologues structures retrieved from the AlphaFold Protein Structure Database superimposed to the human CYP2A6 (PDB ID 2PG6). The main difference to structures reported in figure A is the transmembrane helix (within the red box), which was not relevant for the present study. C) CYP1A2 animal homologues structures obtained via the homology modelling procedure based on Modeller superimposed to the human CYP1A2 (PDB ID 2HI4). D) CYP1A2 animal homologues structures retrieved from the AlphaFold Protein Structure Database superimposed to the human CYP1A2 (PDB ID 2HI4). The main difference to structures reported in figure C is the transmembrane helix (within the red box), which was not relevant for the present study; Figure S5: Percent Identity Matrices (PIMs) for CYP2A6 homologs.

## References

[1] Gotz M.E., Sachse B., Schafer B., Eisenreich A. (2022). Myristicin and Elemicin: Potentially Toxic Alkenylbenzenes in Food. Foods.

[2] Eisenreich A., Gotz M.E., Sachse B., Monien B.H., Herrmann K., Schafer B. (2021). Alkenylbenzenes in Foods: Aspects Impeding the Evaluation of Adverse Health Effects. Foods.

[3] Atkinson R.G. (2018). Phenylpropenes: Occurrence, Distribution, and Biosynthesis in Fruit. J. Agric. Food Chem..

[4] Jeurissen S.M.F., Punt A., Boersma M.G., Bogaards J.J.P., Fiamegos Y.C., Schilter B., van Bladeren P.J., Cnubben N.H.P., Rietjens I. (2007). Human cytochrome p450 enzyme specificity for the bioactivation of estragole and related alkenylbenzenes. Chem. Res. Toxicol..

[5] IARC (1987). IARC Monographs on the Evaluation of the Carcinogenic Risks to Humans. OveraIl Evaluations of Carcinogenicity: An Updating of IARC Monographs.

[6] Bampidis V., Azimonti G., Bastos M.D., Christensen H., Durjava M.F., Kouba M., Lopez-Alonso M., Puente S.L., Marcon F., Mayo B. (2021). Safety and efficacy of a feed additive consisting of a tincture from the bark of Cinnamomum verum J. Presl (cinnamon tincture) for use in all animal species (FEFANA asbl). Efsa J..

[7] Hausner E., Poppenga R.H., Peterson M.E., Talcott P.A. (2013). Chapter 26—Hazards Associated with the Use of Herbal and Other Natural Products. Small Animal Toxicology.

[8] Prinsloo G., Nogemane N., Street R. (2018). The use of plants containing genotoxic carcinogens as foods and medicine. Food Chem. Toxicol..

[9] Siano F., Ghizzoni C., Gionfriddo F., Colombo E., Servillo L., Castaldo D. (2003). Determination of estragole, safrole and eugenol methyl ether in food products. Food Chem..

[10] Bampidis V., Azimonti G., Bastos M.D., Christensen H., Durjava M.F., Kouba M., Lopez-Alonso M., Puente S.L., Marcon F., Mayo B. (2022). Safety and efficacy of a feed additive consisting of an essential oil from *Cinnamomum camphora* (L.) J. Presl (camphor white oil) for use in all animal species (FEFANA asbl). Efsa J..

[11] Bode A.M., Dong Z.G. (2015). Toxic Phytochemicals and Their Potential Risks for Human Cancer. Cancer Prev. Res..

[12] Martati E., Boersma M.G., Spenkelink A., Khadka D.B., van Bladeren P.J., Rietjens I., Punt A. (2012). Physiologically Based Biokinetic (PBBK) Modeling of Safrole Bioactivation and Detoxification in Humans as Compared with Rats. Toxicol. Sci..

[13] Carmichael P.L., Baltazar M.T., Cable S., Cochrane S., Dent M., Li H.Q., Middleton A., Muller I., Reynolds G., Westmoreland C. (2022). Ready for Regulatory Use: NAMs and NGRA for Chemical Safety Assurance. Altex Altern. Anim. Exp..

[14] Itoh T., Takemura H., Shimoi K., Yamamoto K. (2010). A 3D Model of CYP1B1 Explains the Dominant 4-Hydroxylation of Estradiol. J. Chem. Inf. Model..

[15] Dorne J., Cirlini M., Louisse J., Pedroni L., Galaverna G., Dellafiora L. (2022). A Computational Understanding of Inter-Individual Variability in CYP2D6 Activity to Investigate the Impact of Missense Mutations on Ochratoxin A Metabolism. Toxins.

[16] Sridhar J., Goyal N., Liu J.W., Foroozesh M. (2017). Review of Ligand Specificity Factors for CYP1A Subfamily Enzymes from Molecular Modeling Studies Reported to-Date. Molecules.

[17] von Weymarn L.B., Murphy S.E. (2001). Coumarin metabolism by rat esophageal microsomes and cytochrome P450 2A3. Chem. Res. Toxicol..

[18] Papatheodorou I., Moreno P., Manning J., Fuentes A.M.P., George N., Fexova S., Fonseca N.A., Fullgrabe A., Green M., Huang N. (2020). Expression Atlas update: From tissues to single cells. Nucleic Acids Res..

[19] Bastian F.B., Roux J., Niknejad A., Comte A., Costa S.S.F., de Farias T.M., Moretti S., Parmentier G., de Laval V.R., Rosikiewicz M. (2021). The Bgee suite: Integrated curated expression atlas and comparative transcriptomics in animals. Nucleic Acids Res..

[20] Righetti L., Rolli E., Dellafiora L., Galaverna G., Suman M., Bruni R., Dall’Asta C. (2021). Thinking Out of the Box: On the Ability of *Zea mays* L. to Biotrasform Aflatoxin B1 Into Its Modified Forms. Front. Plant Sci..

[21] Altschul S.F., Gish W., Miller W., Myers E.W., Lipman D.J. (1990). Basic local alignment search tool. J. Mol. Biol..

[22] Jeurissen S.M.F., Bogaards J.J.P., Awad H.M., Boersma M.G., Brand W., Fiamegos Y.C., van Beek T.A., Alink G.M., Sudholter E.J.R., Cnubben N.H.P. (2004). Human cytochrome P450 enzyme specificity for bioactivation of safrole to the proximate carcinogen 1′-hydroxysafrole. Chem. Res. Toxicol..

[23] Martati E., Boersma M.G., Spenkelink A., Khadka D.B., Punt A., Vervoort J., van Bladeren P.J., Rietjens I. (2011). Physiologically Based Biokinetic (PBBK) Model for Safrole Bioactivation and Detoxification in Rats. Chem. Res. Toxicol..

[24] Kishida T., Muto S.I., Hayashi M., Tsutsui M., Tanaka S., Murakami M., Kuroda J. (2008). Strain differences in hepatic cytochrome P450 1A and 3A expression between Sprague-Dawley and Wistar rats. J. Toxicol. Sci..

[25] Sayers E.W., Bolton E.E., Brister J.R., Canese K., Chan J., Comeau D.C., Connor R., Funk K., Kelly C., Kim S. (2022). Database resources of the national center for biotechnology information. Nucleic Acids Res..

[26] Vangone A., Schaarschmidt J., Koukos P., Geng C.L., Citro N., Trellet M.E., Xue L.C., Bonvin A. (2019). Large-scale prediction of binding affinity in protein-small ligand complexes: The PRODIGY-LIG web server. Bioinformatics.

[27] Kim S., Chen J., Cheng T.J., Gindulyte A., He J., He S.Q., Li Q.L., Shoemaker B.A., Thiessen P.A., Yu B. (2021). PubChem in 2021: New data content and improved web interfaces. Nucleic Acids Res..

[28] Berman H.M., Westbrook J., Feng Z., Gilliland G., Bhat T.N., Weissig H., Shindyalov I.N., Bourne P.E. (2000). The Protein Data Bank. Nucleic Acids Res..

[29] Sansen S., Yano J.K., Reynald R.L., Schoch G.A., Griffin K.J., Stout C.D., Johnson E.F. (2007). Adaptations for the oxidation of polycyclic aromatic hydrocarbons exhibited by the structure of human P450 1A2. J. Biol. Chem..

[30] Sansen S., Hsu M.H., Stout C.D., Johnson E.F. (2007). Structural insight into the altered substrate specificity of human cytochrome P450 2A6 mutants. Arch. Biochem. Biophys..

[31] Pettersen E.F., Goddard T.D., Huang C.C., Couch G.S., Greenblatt D.M., Meng E.C., Ferrin T.E. (2004). UCSF chimera—A visualization system for exploratory research and analysis. J. Comput. Chem..

[32] Louisse J., Dorne J.L.C.M., Dellafiora L. (2022). Investigating the interaction between organic anion transporter 1 and ochratoxin A: An in silico structural study to depict early molecular events of substrate recruitment and the impact of single point mutations. Toxicol. Lett..

[33] Dellafiora L., Oswald I.P., Dorne J.L., Galaverna G., Battilani P., Dall’Asta C. (2020). An in silico structural approach to characterize human and rainbow trout estrogenicity of mycotoxins: Proof of concept study using zearalenone and alternariol. Food Chem..

[34] Sali A., Blundell T.L. (1993). Comparative protein modeling by satisfaction of spatial restraints. J. Mol. Biol..

[35] Jumper J., Evans R., Pritzel A., Green T., Figurnov M., Ronneberger O., Tunyasuvunakool K., Bates R., Zidek A., Potapenko A. (2021). Highly accurate protein structure prediction with AlphaFold. Nature.

[36] Larkin M.A., Blackshields G., Brown N.P., Chenna R., McGettigan P.A., McWilliam H., Valentin F., Wallace I.M., Wilm A., Lopez R. (2007). Clustal W and clustal X version 2.0. Bioinformatics.

[37] Maldonado-Rojas W., Olivero-Verbel J. (2011). Potential interaction of natural dietary bioactive compounds with COX-2. J. Mol. Graph. Model..

[38] Adasme M.F., Linnemann K.L., Bolz S.N., Kaiser F., Salentin S., Haupt V.J., Schroeder M. (2021). PLIP 2021: Expanding the scope of the protein-ligand interaction profiler to DNA and RNA. Nucleic Acids Res..

[39] Abraham M.J., Murtola T., Schulz R., Páll S., Smith J.C., Hess B., Lindahl E. (2015). GROMACS: High performance molecular simulations through multi-level parallelism from laptops to supercomputers. SoftwareX.

[40] Best R.B., Zhu X., Shim J., Lopes P.E.M., Mittal J., Feig M., MacKerell A.D. (2012). Optimization of the Additive CHARMM All-Atom Protein Force Field Targeting Improved Sampling of the Backbone Φ, Ψ and Side-Chain χ1 and χ2 Dihedral Angles. J. Chem. Theory Comput..

[41] Zhang L., Silva D.A., Yan Y.J., Huang X.H. (2012). Force field development for cofactors in the photosystem II. J. Comput. Chem..

[42] Panneerselvam S., Yesudhas D., Durai P., Anwar M.A., Gosu V., Choi S. (2015). A Combined Molecular Docking/Dynamics Approach to Probe the Binding Mode of Cancer Drugs with Cytochrome P450 3A4. Molecules.

[43] Del Favero G., Mayer R.M., Dellafiora L., Janker L., Niederstaetter L., Dall’Asta C., Gerner C., Marko D. (2020). Structural Similarity with Cholesterol Reveals Crucial Insights into Mechanisms Sustaining the Immunomodulatory Activity of the Mycotoxin Alternariol. Cells.

