# Supplementary material for: A Computational Inter-Species Study on Safrole Phase I Metabolism-Dependent Bioactivation: A Mechanistic Insight into the Study of Possible Differences among Species
Source: Toxins (Basel). 2023 Jan 18;15(2):94. doi: 10.3390/toxins15020094 (PMC9962551; doi:10.3390/toxins15020094)
Supplement: Supplementary file 1 [file toxins-15-00094-s001.zip › toxins-2086787-supplementary.pdf]

Supplementary Materials

A Computational Inter-Species Study on Safrole Phase I Metabolism-Dependent Bioactivation: A Mechanistic Insight into the Study of Possible Differences among Species

Table S1. Expression data for considered CYPs at the liver level in the species under analysis.

**Species**	**CYP2A6 and its homologues**			**CYP1A2**		
	**UniProt** [1]	**Expression Atlas** [2]	**Bgee** [3]	**UniProt** [1]	**Expression Atlas** [2]	**Bgee** [3]
Human	Evidence at protein level	692010 parts per billion	98.57	Evidence at protein level	519668 parts per billion	95.32
Rat	Evidence at transcript level	n.a.	n.a.	Evidence at protein level	n.a.	98.65
Mouse	Evidence at transcript level	37033 parts per billion	91.96	Evidence at protein level	216750 parts per billion	99.16
Cat	Inferred from homology	n.a.	99.5	Evidence at transcript level	n.a.	98.42
Dog	Evidence at transcript level	n.a.	75.43	Evidence at protein level	n.a.	74.07
Pig	Evidence at protein level	n.a.	96.28	Evidence at protein level	n.a.	97.64
Goat	Inferred from homology	n.a.	96.66	Inferred from homology	n.a.	88.84
Rabbit	Evidence at protein level (CYP2A10)	n.a.	99.49	Evidence at protein level	n.a.	99.46
	Evidence at protein level (CYP2A11)	n.a.	n.a.			
Chicken	---	---	---	Evidence at transcript level	n.a.	94.68
Sheep	Evidence at transcript level	n.a.	53.57	n.a.	n.a.	58.23

Note: [1] Information of protein existence according to UniProt database (https://www.uniprot.org); [2] Data at protein level was considered (if any) and the highest value was reported according to Expression Atlas database (https://www.ebi.ac.uk/gxa/home); [3] Expression scores according to Bgee database (https://bgee.org) are reported; "n.d." stands for "data not available".

Table S2. Accession code and percentage identity of animal homologs to human CYP2A6.

Species [1]	Homolog to CYP2A6	Identity % to human CYP2A6
Rat (10116)	P20812 (CYP2A3)	85.38%
Mouse (10090)	P20852 (CYP2A5)	85.59%
Dog (9615)	Q307K8 (CYP2A13)	88.87%
Rabbit (9986)	Q05555 (CYP2A10)	83.69%
	Q05556 (CYP2A11)	83.47%
Pig (9823)	Q8SQ68 (CYP2A19)	86.44%
Goat (9925)	A0A452DNG6 (CYP2A13)	88.35%
Sheep (9940)	F1CGV2 (CYP2A6)	88.54%
Cat (9685)	M3W9T6 (CYP2A13)	86.65%
Chicken (9031)	ND [2]	< 50%

Note: [1] the species taxon ID is reported between brackets; [2] ND stands for data not found, meaning that the identity percentage to the human homolog was lower than 50% at the time of analysis (last database access 8th July 2022).

Table S3. Accession number for animal homolog to human CYP1A2 or CYP2A6.

Species [1]	Homolog to CYP1A2	Homolog to CYP2A6
Rat (10116)	P04799	P20812 (CYP2A3)
Mouse (10090)	P00186	P20852 (CYP2A5)
Dog (9615)	P56592	Q307K8 (CYP2A13)
Rabbit (9986)	P00187	Q05555 (CYP2A10)
		Q05556 (CYP2A11)
Pig (9823)	F1SJ26	Q8SQ68 (CYP2A19)
Goat (9925)	A0A452EFF2	A0A452DNG6 (CYP2A13)
Sheep (9940)	XP027812985	F1CGV2 (CYP2A6)
Cat (9685)	Q5KQT6	M3W9T6 (CYP2A13)
Chicken (9031)	Q01741	ND [2]

Note: [1] the species taxon ID is reported between brackets; [2] ND stands for data not found, meaning that the identity percentage to the human homolog was lower than 50% at the time of analysis (last database access 8th July 2022).

Table S4. Structural comparison between the CYP2A6 homology modelling structures obtained using Modeller and the AlphaFold Protein Structure Database.

**Species** [1]	**Homolog to CYP2A6**	**RMSD (Å)** [2]	**RMSD (Å)** [3]	**RMSD (Å)** [4]
Rat (10116)	P20812 (CYP2A3)	0.27	0.32	0.35
Mouse (10090)	P20852 (CYP2A5)	0.28	0.33	0.37
Dog (9615)	Q307K8 (CYP2A13)	0.28	0.33	0.62
Rabbit (9986)	Q05555 (CYP2A10)	0.27	0.42	0.42
	Q05556 (CYP2A11)	0.27	0.42	0.42
Pig (9823)	Q8SQ68 (CYP2A19)	0.27	0.31	0.33
Goat (9925)	A0A452DNG6 (CYP2A13)	0.28	0.31	0.34
Sheep (9940)	F1CGV2 (CYP2A6)	0.28	0.31	0.33
Cat (9685)	M3W9T6 (CYP2A13)	0.27	0.32	0.34

Note: [1] species taxon ID is reported between brackets; [2] RMSD computed superimposing the structure obtained via homology modelling using Modeller and the human CYP2A6 (PDB ID 2PG6); [3] RMSD computed superimposing the structure obtained from the AlphaFold Protein Structure Database and the human CYP2A6 (PDB ID 2PG6); [4] RMSD computed superimposing the structure obtained via Homology modelling and the one obtained on the AlphaFold Protein Structure Database.

Table S5. Structural comparison between the CYP1A2 homology modelling structures obtained using Modeller and the AlphaFold Protein Structure Database.

**Species** [1]	**RMSD (Å)** [2]	**RMSD (Å)** [3]	**RMSD (Å)** [4]
Rat (10116)	0.33	0.40	0.41
Mouse (10090)	0.30	0.43	0.44
Dog (9615)	0.17	0.38	0.39
Rabbit (9986)	0.17	0.44	0.46
Chicken (9031)	0.25	0.57	0.58
Pig (9823)	0.16	0.39	0.40
Goat (9925)	0.15	0.38	0.38
Sheep (9940)	0.17	0.73*	0.72*
Cat (9685)	0.18	0.48	0.63

Note: [1] species taxon ID is reported between brackets; [2] RMSD computed superimposing the structure obtained via homology modelling using Modeller and the human CYP1A2 (PDB ID 2HI4); [3] RMSD computed superimposing the structure obtained from the AlphaFold Protein Structure Database and the human CYP1A2 (PDB ID 2HI4); [4] RMSD computed superimposing the structure obtained via homology modelling using Modeller and the one obtained on the AlphaFold Protein Structure Database.* The sheep CYP1A2 was not available on the AlphaFold Protein Structure Database and was then computed via the AlphaFold2 Colab notebook using default parameter (https://colab.research.google.com/github/sokrypton/ColabFold/blob/main/AlphaFold2.ipynb).

Table S6. Average predicted binding energy of the complexes under analysis

Molecule	Species CYP	ΔG (Kcal/mol) [1]	Number of clusters [2]
Estragole	Human 1A2	-7.20	1
	Human 2A6	-7.00	1
Coumarin	Human 2A6	-7.10	1
	Rat 2A3	-7.00	1
Safrole	Human 1A2	-7.30	1
	Human 2A6	-7.20	1
	Cat 1A2	-7.40	1
	Cat 2A13	-7.25 ± 0.05 *	3
	Chicken 1A2	-7.30	1
	Dog 1A2	-7.30	1
	Dog 2A13	-7.30	1
	Goat 1A2	-7.30	1
	Goat 2A13	-7.10	1
	Mouse 1A2	-7.20	1
	Mouse 2A5	-7.20	1
	Pig 1A2	-7.40	1
	Pig 2A19	-7.20	1
	Rat 1A2	-7.20	1
	Rat 2A3	-7.30	1
	Rabbit 1A2	-7.30	1
	Rabbit 2A10	-7.30	1
	Rabbit 2A11	-7.30	1
	Sheep 1A2	-7.20	1
	Sheep 2A6	-7.20	1

Note: [1] Binding energy computed on the PRODIGY web application on the cluster's representative complex (https://wenmr.science.uu.nl/prodigy/lig); [2] Number of clusters retrieved along the whole molecular dynamicssimulation; * The value reported represents the average value of each representative cluster.

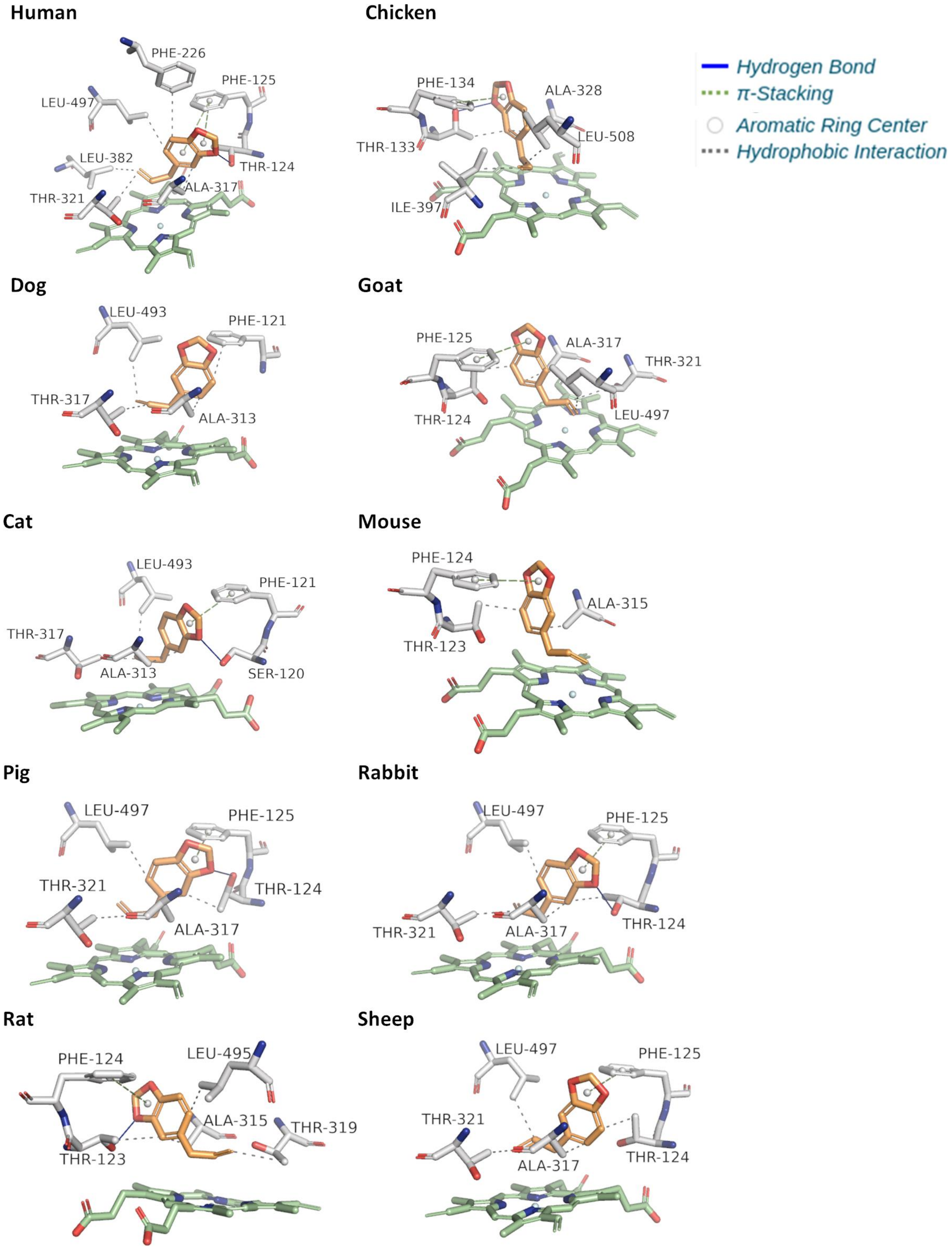

Figure S1. Binding architecture of safrole in complex with human CYP1A2 and its animal homologs. Protein is shown in white sticks, ligand in yellow and heme in green.

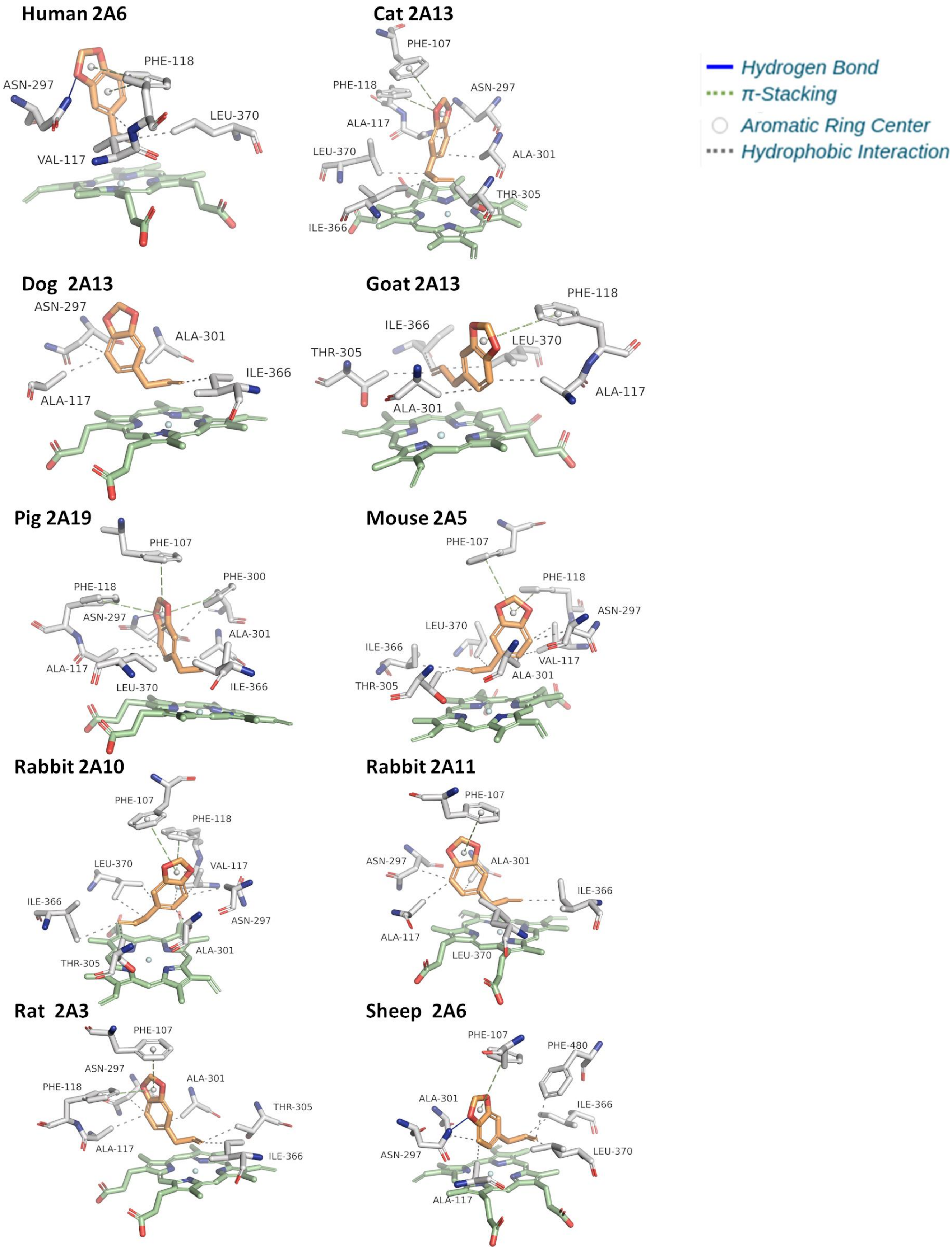

Figure S2. Binding architecture of safrole in complex with human CYP2A6 and its animal homologs. Protein is shown in white sticks, ligand in yellow and heme in green.

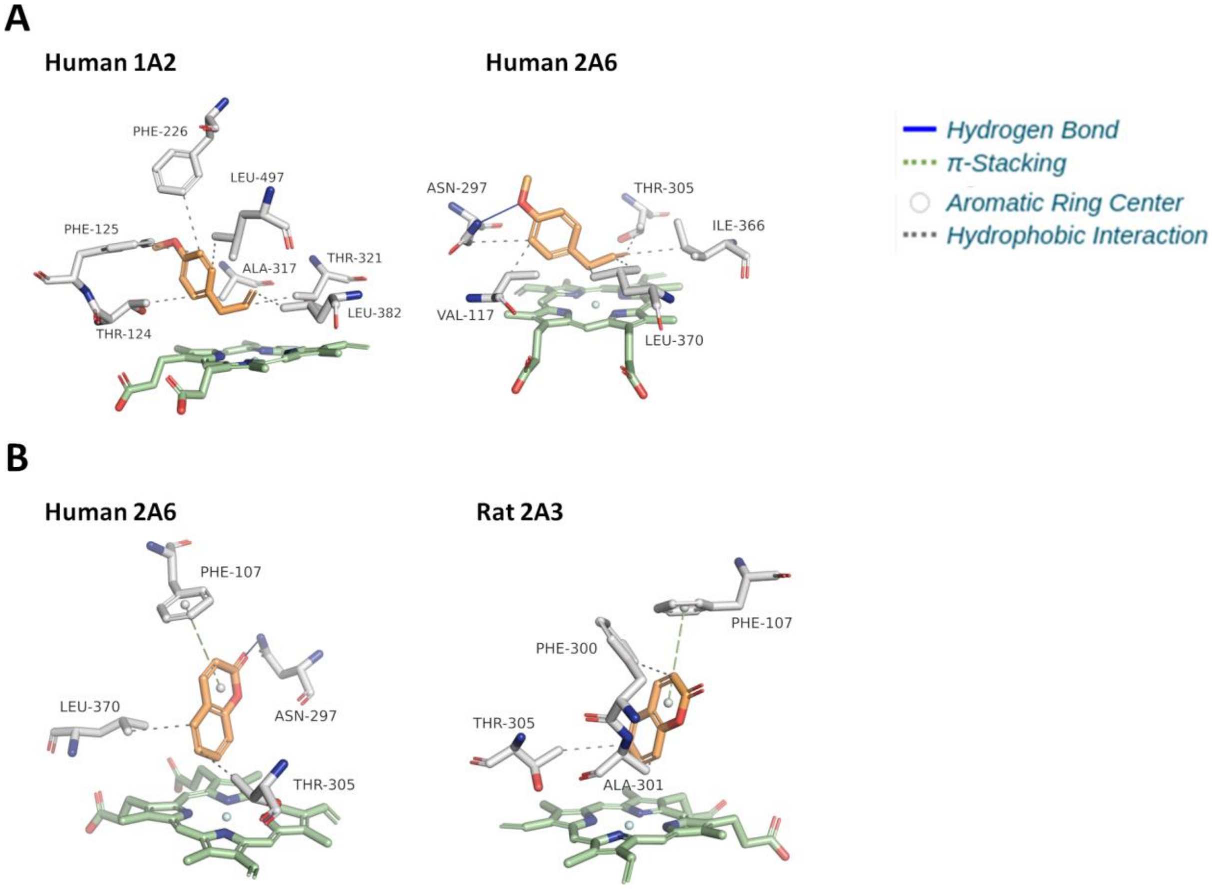

Figure S3. Binding architecture of estragole within human CYP1A2 and CYP2A6 (**A**) and coumarin in human CYP2A6 and rat CYP2A3 (**B**). Protein is shown in white sticks, ligand in yellow and heme in green.

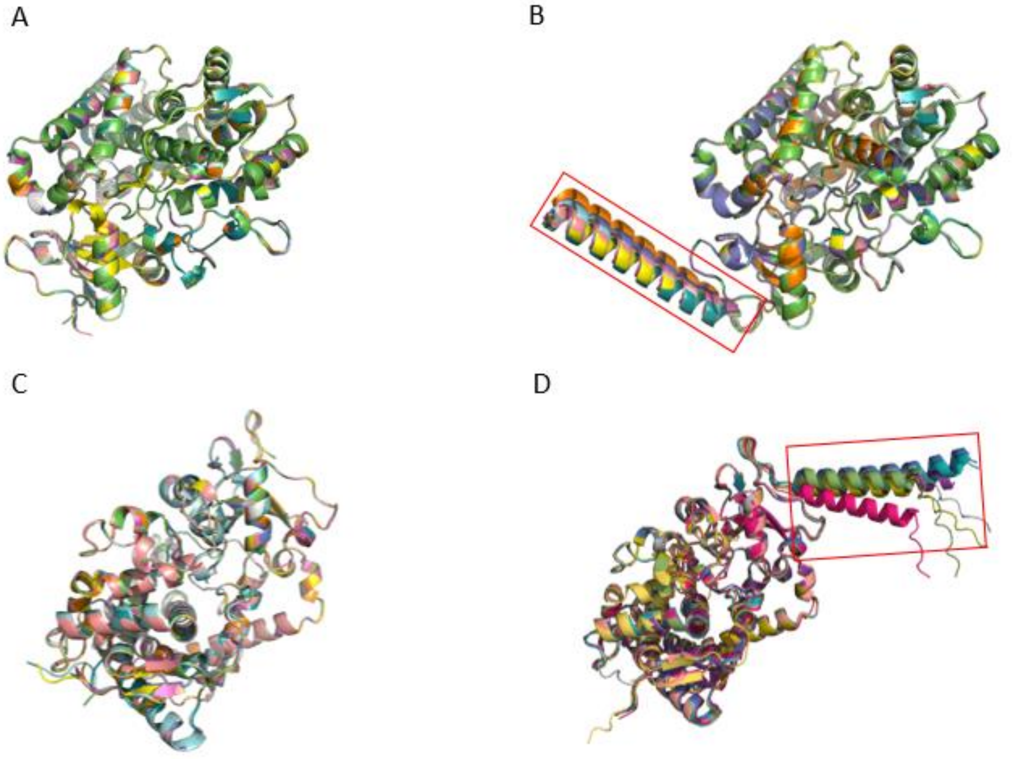

Figure S4. Structural alignment of the animals' CYPs homologues over the human CYP2A6 (PDB ID 2PG6) and CYP1A2 (PDB ID 2HI4). **A**) CYP2A6 animal homologues structures obtained via the homology modelling procedure based on Modeller superimposed to the human CYP2A6 (PDB ID 2PG6). **B**) CYP2A6 animal homologues structures retrieved from the AlphaFold Protein Structure Database superimposed to the human CYP2A6 (PDB ID 2PG6). The main difference to structures reported in figure A is the transmembrane helix (within the red box), which was not relevant for the present study. **C**) CYP1A2 animal homologues structures obtained via the homology modelling procedure based on Modeller superimposed to the human CYP1A2 (PDB ID 2HI4). **D**) CYP1A2 animal homologues structures retrieved from the AlphaFold Protein Structure Database superimposed to the human CYP1A2 (PDB ID 2HI4). The main difference to structures reported in figure C is the transmembrane helix (within the red box), which was not relevant for the present study.

```
#
#
# CYP2A6 Percent Identity  Matrix - created by Clustal2.1
#
#

     1: Human-2a6               100.00   83.69   83.47   85.59   85.38   86.65   88.77   86.44   88.54   88.35
     2: Rabbit-2a10              83.69  100.00   98.38   86.23   86.03   85.83   87.45   87.85   89.05   88.87
     3: Rabbit-2a11              83.47   98.38  100.00   86.44   86.64   86.44   88.06   88.66   89.66   89.47
     4: Mouse-2a5                85.59   86.23   86.44  100.00   96.15   86.23   89.07   89.07   89.25   89.27
     5: Rat-2a3                  85.38   86.03   86.64   96.15  100.00   87.25   90.08   88.87   89.86   89.68
     6: Cat-2a13                 86.65   85.83   86.44   86.23   87.25  100.00   94.13   91.30   91.89   92.11
     7: Dog-2a13                 88.77   87.45   88.06   89.07   90.08   94.13  100.00   92.11   93.31   93.12
     8: Pig-2a19                 86.44   87.85   88.66   89.07   88.87   91.30   92.11  100.00   93.10   93.32
     9: Sheep-2a6                88.54   89.05   89.66   89.25   89.86   91.89   93.31   93.10  100.00   99.39
    10: Goat-2a13                88.35   88.87   89.47   89.27   89.68   92.11   93.12   93.32   99.39  100.00
```

Figure S5. Percent Identity Matrices (PIMs) for CYP2A6 homologs.
